# Neuropsychological Assessment Using Portable Automated Rapid Testing in Cognitively Healthy Older Adults: Test-Retest Reliability Study

**DOI:** 10.2196/90646

**Published:** 2026-09-23

**Authors:** Quentin Coppola, Lakshmi Kannan, Audrey Carrillo, Susanne M Jaeggi, Aaron R Seitz

**Affiliations:** 1 Department of Psychology College of Science Northeastern University Boston, MA United States; 2 Brain Game Center for Mental Fitness and Well-Being Boston, MA United States

**Keywords:** aging, digital health, feasibility, neuropsychology, reliability, remote assessment

## Abstract

**Background:**

Remote, scalable cognitive assessment could improve detection and longitudinal monitoring of age-related cognitive change, but few studies have validated comprehensive digital neuropsychological batteries administered entirely at home.

**Objective:**

This study aims to evaluate the feasibility, acceptability, and short-term test-retest reliability of a remotely delivered digital neuropsychological battery (portable automated rapid testing [PART]) in cognitively healthy older adults.

**Methods:**

We screened 82 English-speaking, cognitively healthy older adults aged 50 to 85 years and mailed configured tablets to participants. Researchers remotely administered a battery of neuropsychological assessments spanning language fluency (Boston Naming Test, verbal fluency: letter “F,” supermarket items, and animals), memory (verbal paired associates, word list recall, and logical memory recall), praxis memory (clock drawing and constructional praxis [CP]), and executive functioning (trail making test [TMT]) using the PART app twice, approximately 1 month apart. Task comfortability was summarized with a comfort index based on participants’ self-reports after completing each task. Reliability analyses included paired *t* tests to detect group-level shifts in performance, Pearson correlations for short-term stability, and Bland-Altman limits of agreement (LoA) to quantify bias and within-person variability. Performance across all tasks was also compared to comparative samples from large normative studies using equivalent paper-and-pencil versions of these tasks.

**Results:**

A total of 72 participants completed the first time point (T1), 64 completed the second time point (T2), and the analytic sample comprised a total of 63 participants. Overall comfort was high across sessions (mean comfort index was 88% at T1 and 84% at T2). Memory and executive function measures showed moderate test-retest correlations (*r*=0.39-0.73), while clock drawing and CP showed low or nonsignificant associations, likely due to ceiling effects. Paired tests indicated small but significant practice effects for verbal paired associates (t_62_=−3.10; *P*=.003) and word list (t_57_=−2.50; *P*=.02), with substantial practice effects for logical memory (story A: t_56_=−2.95; *P*=.005) and TMT part A (t_50_=−3.97; *P*<.001) and part B (t_42_=−2.05; *P*=.05). Bland-Altman analyses revealed minimal mean bias for many tasks but notably wide LoA for TMT (part A: LoA=−44.7 to 29.2 seconds; part B: LoA=−78.1 to 57.9 seconds). Importantly, in descriptive comparisons, our sample generally overlapped within ±1 SD of the medians in comparative paper-and-pencil normative samples.

**Conclusions:**

PART was well tolerated among healthy older adults and reproduces group-level normative patterns with moderate short-term reliability for several conventional measures. However, nontrivial within-person variability, practice effects, ceiling effects, and wide LoAs for some measures (eg, TMT, CP, and clock drawing) limit interpretation of individual change. Future work should validate alternative digital outcome metrics (eg, stylus interactions and speech recordings), extend sampling to more diverse and lower-education cohorts, and evaluate more ecologically valid designs to improve sensitivity for detecting meaningful within-person cognitive fluctuations.

## Introduction

Globally, the number of older adults is projected to grow substantially over the next 2 decades [1]. Such a significant demographic shift brings increased incidence rates of age-related cognitive decline, mild cognitive impairment, and dementia, highlighting the need for more accessible, efficient, and scalable cognitive screening methods to keep pace with the identification of clinically meaningful decline and fluctuations of cognition [2,3]. While conventional in-clinic, paper-based tests have been the standard form of screening and testing, the recent advent of reliable and user-friendly digital technologies has expanded opportunities to move cognitive evaluations into digital paradigms, increasing in complexity and dimensionality while providing opportunities for richer exploration of behavior [4]. These technologies also look to break systemic and environmental barriers that limit access to underserved populations in both cognitive research and vital health care monitoring [5]. Additionally, digital assessment platforms can automate scoring and data storage, thereby reducing administrative burden and cost, while also providing an opportunity to reach larger populations through remote testing [6]. Transitioning to digital neuropsychological assessment has strong potential to bolster the capacity to detect, monitor, and address cognitive changes in older adults by enabling scalable, longitudinal monitoring that can complement traditional clinic-based assessment and advance research at the population level.

A growing body of evidence supports the digital adaptation of well-established paper-and-pencil neuropsychological tasks and cognitive screening batteries [7-11]. Cognitive screeners, such as the Montreal Cognitive Assessment, have been shown to be reliable in digital form [12]. Digital versions of standard neuropsychological tests, such as the Trail Making Test (TMT), a measure of executive functioning, have shown good correspondence to paper-and-pencil counterparts [13-15]. Similarly, the Clock Drawing Test, a longstanding yet quickly administered dementia screener capturing executive and visuospatial function, has been demonstrated to be a reliable and valid tool when converted to digital modes [16]. These 3 tasks are just a few examples of many tests that have been successfully digitized for use via mobile smartphones or tablets, each demonstrating acceptable sensitivity and specificity in detecting cognitive impairment. However, a broader effort to integrate these digitized assessments into comprehensive digital batteries has emerged, with several platforms not available for use in older adult populations.

Tablet-based platforms for neuropsychological testing, such as Cogstate C3 [17], Tablet-Based Cognitive Assessment Tool (TabCAT) [18], or the Cambridge Neuropsychological Test Automated Battery (CANTAB) [19], offer well-validated, semi–self-administered testing in older adult populations with and without cognitive decline. However, these commonly used systems are narrow in their scope and require proprietary licensing, constraining the range of constructs they can measure and who can collect data using them. In the same vein, remote batteries specifically validated for older adult cognitive monitoring, such as the Boston Remote Assessment for Neurocognitive Health [20] and similar mobile and web-based platforms, focus on a limited number of tasks that primarily function as screeners for clinical cognitive decline. The National Institutes of Health (NIH) Toolbox Cognition Battery [21] offers more tasks for a wider number of age groups; however, a number of studies have identified issues in these tasks’ sensitivity compared to alternative digital testing platforms, especially in older adult populations [22,23]. Critically, all these testing systems suffer from limited configurability in both the tasks they can administer and the degree to which researchers can customize these tasks to fit their research needs, which can be constraining to basic research.

Understanding how reliably these comprehensive assessments perform over time is critical, particularly given that cognitive performance in older adults can fluctuate due to transient factors such as mood, fatigue, and comorbid clinical conditions [24]. Evaluating the stability of scores between 2 closely administered assessments can help to clarify whether changes reflect real cognitive shifts or measurement inconsistencies. Digital tools may be helpful in reliably detecting and modeling these fluctuations, which may be critical for capturing small changes that precede clinical decline or for characterizing specific cognitive phenotypes [25]. Ambulatory platforms such as the Mobile Monitoring of Cognitive Change (M2C2) [26] and the Ambulatory Research in Cognition (ARC) app [27] offer brief, frequently repeated assessments delivered on participants’ own smartphones to capture intraindividual fluctuations in cognition [28]. Although these platforms offer strong ecological validity, their brief, single-domain assessments are not designed to capture the full range of constructs that comprehensive clinical neuropsychological batteries traditionally assess. If digitally adapted assessments that expand on these narrow measurement targets exhibit robust reliability over temporal intervals while accounting for possible fluctuations in individual differences, they may prove ideal for continuous longitudinal monitoring of cognitive trajectories in clinical trials, primary care, and at-home settings. Conversely, evidence of inconsistent scores would underline the need for further technological refinements, novel analysis models to account for this inconsistency, or additional training sessions for less reliable performers to ensure accuracy and participant adherence and acceptance.

Moreover, the transition from paper-based assessments to digital ones requires considerations beyond the cognitive demands the task provides. Accordingly, digital assessments must implement an intuitive and tailored design, require minimal technological expertise to use, and provide adequate instructions to mitigate confounding variables. Establishing the usability and acceptability of these tools among older users is a critical step before they can be fully integrated into regular clinical practice. Several studies have mitigated these confounds by requiring psychometricians or study administrators to assist in person with administration [5,14]. However, there is still a need for platforms that can accommodate diverse testing use cases without sacrificing the usability of the tasks themselves or the underlying cognitive construct the task is trying to measure.

Despite numerous advances, the current literature lacks sufficient studies examining the reliability and feasibility of comprehensive digital neuropsychological testing in remote settings. Our contribution to this emerging field is to evaluate digital versions of traditional paper-and-pencil neuropsychological tests, examining feasibility and repeatability in diverse clinical and research contexts [29]. To achieve this, our laboratory has developed the portable automated rapid testing (PART) [30] platform, a mobile app that facilitates remote data collection without compromising the breadth and depth of the testing schema or the validity of tasks when compared to in-laboratory testing. PART is a freely accessible digital app available via the iOS App Store and the Google Play Store. Where PART builds upon the digital assessment platforms currently available is that it is highly configurable in terms of the parameters of individual tests (eg, stimuli and timing parameters) and the ability to create testing batteries that can combine assessments of different modalities (hearing [31,32], vision [33], executive functions [34], attentional control [35], decision making [36], gamified interventions [37], and, in the current paper, neuropsychological tests). In turn, this allows researchers to have a system that can be used for both clinical evaluation and basic research.

The current work aims to examine test-retest reliability and participant-reported usability of a standard neuropsychological battery within PART administered twice across a 1-month interval. The tasks assess verbal fluency (VF), memory encoding and recall, constructional praxis (CP), and executive functioning. By analyzing any changes in performance, user engagement, acceptability, and data quality, we seek to determine how well older adults adapt to digital neuropsychological testing and whether these tools can consistently capture cognitive data over short intervals.

## Methods

### Ethical Considerations

Study procedures were approved by the University of California (UC), Riverside Institutional Review Board (IRB; number HS-20-177). Informed consent was obtained over the phone during screening, and participants received an online version of the consent form, where they provided a digital signature. Study participants were assigned a subject identification number, and all data were deidentified prior to analysis. Participants received a US $150 Amazon gift card for their participation: US $75 for completing the first time point of the study and US $75 for completing the second.

### Participants

Cognitively healthy older adults aged 50 years or older residing in Southern California were recruited for the study. Data were collected from September 2023 to July 2024. Participants were recruited through online advertisements via ClinicalTrials.gov, university mailing lists, including the UC Riverside Aging Database and the UC Irvine Cradle-to-Career Registry, and flyers posted in local community centers. Potential participants filled out a Qualtrics survey to determine preliminary eligibility (ie, they were an older adult) and then received the phone screen. If a participant was unable to be reached for the phone screen after 3 attempts via calls or email, depending on their preference, they were deemed nonresponsive and removed from the potential subject pool. To be included in the study, participants had to be able to understand and speak English to follow study procedures and have no self-reported speech, hearing, psychological, or neurological impairments that would interfere with their ability to provide informed consent or participate in the study. Exclusion criteria included a formal diagnosis of dementia or other neurological disease, including mild cognitive impairment, determined by self-report from participants and confirmed by a total score below 17 on the Montreal Cognitive Assessment–Blind/Telephone (T-MoCA) [38], a physical disability that would impede training procedures, a mental illness requiring treatment, and/or significant absences during the study timeline. All screening and task administration were conducted by research staff who received training on all neuropsychological tests as well as the T-MoCA and had practiced on other research personnel before coordinating any data collection. Screening and sessions were scheduled with participants’ preferred schedule during regular business hours (9 AM-5 PM Pacific Standard Time, Monday-Friday).

### Procedures

Participants were shipped study equipment, including a calibrated Microsoft Surface Go 2 or Surface Go 3 tablet (Microsoft Corporation), Sennheiser HD 280 Pro headphones (Sennheiser electronic GmbH & Co KG), and detailed written instructions on how to use the tablet. Participants completed demographic surveys through Qualtrics (Qualtrics LLC), as well as auditory and neuropsychological assessments across 2 time points that were administered approximately 1 month apart. Each time point consisted of 4 separate experimental sessions, each lasting approximately 15 to 60 minutes, scheduled across approximately 1 week. Participants completed a total of 8 sessions across both time points, and the relevant cognitive data presented in the current work were from a single experimental session at each of those time points. The first time point (T1) took place within 1 to 2 weeks following consent, whereas the second time point (T2) repeated all measures approximately 30 to 45 days later. All experimental sessions were conducted remotely via Zoom (Zoom Communications, Inc) by a trained research administrator, who instructed the participant on how to use the tablet and provided instructions for each task across both sessions. Data from all tasks were collected digitally through the PART app [30]. Participants were asked to complete testing in a quiet location free of distractions, using the provided headphones for the Zoom call.

### Tasks

Our battery consisted of well-validated neuropsychological tests spanning major cognitive domains affected in cognitive aging and neurocognitive decline (eg, dementia). These included language/fluency, CP, memory, and executive functioning. These tests represent the core of widely used test batteries (eg, Consortium to Establish a Registry for Alzheimer’s Disease [CERAD] [39] and Adult Changes in Thought study [40]) and were chosen to allow for comparisons to published normative data. Importantly, all tasks maintained the same instructions and, if applicable, stimuli across both T1 and T2. For all manually scored tasks, including clock drawing, CP, logical memory, verbal paired associates, word list recall, and VF, scoring was conducted by trained research administrators who, upon reaching an inter-rater reliability of at least 90% with a senior investigator using practice data, were assigned random rating assignments across participants, tasks, and time points.

### Subjective Task Comfortability

At the end of each task, participants provided comfort levels via Qualtrics surveys collected by the study administrator. Specifically, after each task, participants were asked the following: “Were you comfortable with the pace and difficulty of the task?” Their responses were categorized into 4 groups: “yes,” “maybe,” “no,” and “no response,” with missing, blank, or uninterpretable responses coded as “no response.” A comfort index was computed for each task by taking the frequency of the “yes” responses and adding half the frequency of “maybe” responses (comfort index = yes + 0.5 × maybe). Tasks with higher comfort index values were rated as more comfortable, whereas tasks with lower indices were considered less comfortable.

### Language Fluency

#### VF

VF was a language fluency task designed after the Controlled Oral Word Association Task [41]. Participants were presented with a screen asking them to listen to the administrator while the administrator asked them to verbally name all the items that belonged in a prompted category. Participants had 60 seconds to do so. This procedure was repeated 3 times, with the categories being “animals,” “supermarket items,” and words beginning with the letter “F.” The dependent variable was the total number of correct items in the category, excluding rule violations (ie, items named that did not belong to the specified category) and repeated items.

#### Boston Naming Test

Participants were presented with a series of 23 black-and-white line drawings of objects that were drawn from the original subset of the Boston Naming Test (BNT) [42]. This set consisted of 15 items recommended by CERAD plus 8 other items from revised versions of the test [39]. Each drawing was presented individually, and participants were given 10 seconds to name it verbally. No cues were given to participants. The dependent variable was the number of images correctly named in the allotted time.

### Memory

#### Logical Memory

This task was based on the Logical Memory subtest of the Wechsler Memory Scale-Revised IV [43] and comprised an immediate recall portion and a delayed recall portion. Participants were asked to listen to a story and remember it to the best of their ability. A prerecorded story was then played to the participant, lasting around 30 seconds. Immediately after the presentation, the participants were asked to repeat the story verbally. This process was repeated for 2 different stories of similar lengths, one after another (story A and story B). Later in the battery, participants were similarly asked to recall all the elements of both story A and story B, separately. The dependent variable was the number of story items correctly identified for immediate recall and delayed recall.

#### Verbal Paired Associates

Verbal paired associates (VPA) learning was assessed using an adapted version of the Wechsler Memory Scale III [44]. Participants studied 8 word pairs over 3 successive learning sets. Each training set began when the participant tapped a button on the tablet, triggering a prerecorded audio recording of the word pair. Participants were only exposed to the audio of the word pairs. After each training set, participants completed an immediate recall test where the first word of each pair was presented on the screen and audibly, after which they were asked to verbally provide the corresponding word. Later in the battery, a delayed recall test was administered. During this test, the first word of each of the 8 word pairs was presented (audio only), and participants verbally recalled the matching word in the pair. The primary outcome measure was the total number of correctly recalled word pairs on the delayed test.

#### Word List Memory Recall

This task was adapted from the CERAD Word List Memory [45,46]. This task had a learning portion and a delayed recall. First, learning involved the participant being presented with 10 words sequentially for 3 trials, with each trial randomizing the order of the words. During learning, participants were asked to verbally repeat each word they saw before proceeding to the next. After each trial, participants were immediately asked to recall the words from the list. Approximately 5 minutes later in the battery, after the CP task, participants conducted a delayed recall where they were asked to recall as many of the 10 words as they could remember, verbally. The dependent variable was the number of correctly identified words out of 10.

### Praxis Memory

#### Clock Drawing

Participants were shown a blank screen on the tablet and were instructed by the administrator to draw the face of a clock large enough to include all the numbers on it. Once they were finished, the participants were asked to draw the time “20 to 4.” The drawings were scored by administrators using a scale from 1 to 10 [47].

#### CP

This task was based on the CERAD neuropsychological battery’s CP task and included both immediate copy and delayed recall [48]. In the copy portion of this task, participants were asked to draw with their stylus, one at a time, figures that appeared on their screen. These figures included a circle, a diamond, overlapping rectangles, and a Necker cube (ie, simple 2D wireframe drawing of a cube). Participants were not informed about the later recall task. Later in the battery, participants were asked to recall and draw each shape without any reference. For both copy and delayed recall, each figure was evaluated on size, distortion, and completeness. The total scores were the summed scores of each figure, ranging from 0 to 11.

### Executive Functioning: TMT

We administered 2 versions of the TMT, a paper version and a digital version, across both time points. The TMT was selected for both paper and digital administration because (1) it is a stylus-based executive function task where mode of administration (paper vs tablet) may more meaningfully affect performance [15,49,50] and (2) the paper version could serve as a within-study reference standard given the absence of equivalent norms, allowing comparison of digital performance against the well-validated paper version in the same participants. Their administration was counterbalanced such that half of the cohort completed the digital version first and vice versa. Both versions had 2 parts, A and B, wherein, in part A, participants had to connect circles containing numbers from 1 to 25 in ascending order as fast as they could. If they made a mistake, they had to begin from the last circle and continue in the correct order. For part B, participants had the same number of circles with both ascending letters and numbers and were asked to alternate connecting circles with numbers and circles with letters (eg, 1, A, 2, B...). The digital version was programmed to have the same number of circles as the paper version for both parts A and B, as well as replicate the same size (8 inch × 11 inch) that was provided for the paper version. The outcome measure for both parts A and B for the paper and digital versions was the total draw time, which refers to the total elapsed time from the first mark to completion of the last connection, including any time spent correcting errors.

### Statistical Analyses

Our goal was to assess whether each task’s group-level performance fell within the range of comparable norming studies, exhibited limited bias, correlated with itself, and was generally tolerable across both time points. We explored both random and systematic variability through (1) Pearson correlations, allowing evaluation of within-subject consistency while adjusting for session-related variance; (2) 2-tailed paired samples *t* tests, which informed us of the presence of any systematic session effects; and (3) limits of agreement (LoA) analysis, which quantified absolute agreement, within-person variability, and systematic bias [51]. Further, we examined participants’ comfort with these tasks to assess how tolerable they were by quantifying their responses after each task. Normative data for many of our tasks were jointly aggregated from the CERAD [46] and the Cache County Memory Study [52], both of which have been used as comparative norms for healthy older adults aged 56 years and older in large-scale cohort studies (eg, Framingham Heart Study [53], Adult Changes in Thought Study [40], Mayo’s Older American Normative Studies [54]). Notably, these studies have implemented the tasks presented here in paper form. Further, these comparisons serve as descriptive and illustrative group-level performance distributions, rather than serving as an inferential claim of normative equivalence. All statistical tests used an α of .05, and all analyses were conducted using R (version 4.3.3; R Core Team) [55].

## Results

### Feasibility

A total of 82 English-speaking adults who expressed interest were screened and consented by phone. Eight participants were lost before baseline, leaving 74 who began the first time point (T1). In T1, a total of 2 participants were excluded due to an administrative software error that resulted in more than 50% data loss. Of these 72 who completed T1, another 2 were lost to follow-up, leaving 70 participants at the second time point (T2). Six participants were excluded due to more than 50% unusable data due to administration issues with incorrect configurations of PART by researchers, resulting in 64 participants with usable T2 data. Of these, 1 participant had more than 70% unusable data across both sessions (including negative draw time values for TMT due to a software glitch) and was excluded, yielding a final analytic sample of 63 participants. We used all available comfort data, excluding 9 participants’ comfort data from T1 and 11 participants’ comfort data from T2 due to erroneous coding by research administrators, resulting in either errant reporting (ie, unclear explanations of participant responses to the yes or no comfort questions) or no recorded participant responses to comfort questions at all. In our final analytic sample, fewer than 4% of all data were missing completely at random (MCAR; Little’s MCAR: *χ*^2^_828_=842; *P*=.36). Missingness and exclusions are shown in Figure S1 in Multimedia Appendix 1. Participants had, on average, 43 days in between assessments with an SD of 13 days. Testing for T1 took an average of 78 minutes with an SD of 31 minutes. Feasibility was generally high across waves: 97.3% (72/74) of participants who began T1 were successfully included in analyses. Among those who completed T1, 97.2% (70/72) returned for T2. Of participants who completed T2, 91.4% (64/70) provided usable data. From initial screening to completion of both time points, the overall attrition rate was 23.2% (19/82).

### Participants

The final analytical sample (n=63) had a mean age of 70.4 (SD 6.6; range 58-84) years, was 77% (49/63) female-identifying, and highly educated (mean 16.6, SD 4.0 years of education). The sample self-reported race/ethnicity as 69% (44/63) White, 14% (9/63) Black or African American, 5% (3/63) Asian, more than one race 5% (3/63), and 7% (4/63) other or undeclared, with 11% (7/63) of the total sample identifying as Hispanic/Latino. Results from the *t* tests, Pearson correlations, bias, and LoA are provided in Table 1.

**Table 1:** Summary statistics, 2-tailed paired sample t tests, Pearson correlations, and Bland-Altman analysis of digital assessments across time points. Bias=mean (T1-T2), where lower bias values mean that on average, participants scored higher during T2 compared with T1.

Task name (participants)	T1, median (SD)	T2, median (SD)	*t* test (*df*); *P* value	*r*; *P* value	Bias (LoA^a^)
VF^b^: animals_(total)_ (n=60)	23 (6.0)	22 (7.3)	1.76 (59); .08	0.5; <.001	1.5 (–11.5 to 14.6)
VF: supermarket_(total)_ (n=63)	27 (6.4)	26 (6.4)	0.01 (62); >.99	0.73; <.001	0.0 (–9.4 to 9.4)
VF: F-words_(total)_ (n=63)	13 (4.5)	15 (4.6)	–1.73 (62); .09	0.71; <.001	–0.8 (–7.6 to 6.1)
Boston naming_(score)_ (n=62)	22 (1.5)	22 (1.5)	–0.21 (61); .84	0.67; <.001	0.0 (–2.4 to 2.4)
LM^c^: story A_(score)_ (n=57)	7 (5.3)	10 (4.7)	–2.95 (56); .005	0.39; .003	–2.1 (–12.9 to 8.6)
LM: story B_(score)_ (n=61)	8 (4.9)	10 (4.5)	–2.19 (60); .03	0.39; <.001	–1.5 (–11.8 to 8.9)
VPA^d^: recall_(total)_ (n=63)	6 (1.4)	7 (1.7)	–3.1 (62); .003	0.60; <.001	–0.7 (–3.9 to 2.6)
WL^e^: recall_(total)_ (n=58)	8 (2.1)	8 (1.6)	–2.5 (57); .02	0.65; <.001	–0.5 (–3.4 to 2.4)
Clock Drawing_(score)_ (n=63)	8 (2.2)	9 (2.1)	–0.99 (62); .33	0.04; .76	–0.4 (–6.1 to 5.4)
CP^f^: circle_(score)_ (n=59)	2 (0.6)	2 (0.3)	–2.01 (58); .05	0.31; .02	–0.2 (–1.3 to 1.0)
CP: diamond_(score)_ (n=63)	3 (1.0)	3 (1.1)	–0.17 (62); .87	–0.03; .83	0.0 (–3.0 to 2.9)
CP: Rectangles_(score)_ (n=61)	2 (0.88)	2 (0.85)	–1.01 (60); .32	0.18; .16	–0.1 (–2.4 to 2.1)
CP: Cube_(score)_ (n=63)	3 (1.4)	3 (1.5)	–1.13 (62); .26	0.24; .06	–0.3 (–3.7 to 3.2)
CP: total recall_(score)_ (n=61)	9 (2.98)	9 (2.89)	–0.95 (60); .34	0.13; .34	–0.5 (–8.1 to 7.2)
TMT-A^g^_(total draw time)_ (n=51)	33.3 (10.4)	38.5 (17.8)	–3.97 (50); <.001	0.66; <.001	–7.8 (–44.7 to 29.2)
TMT-B^h^_(total draw time)_ (n=43)	66.4 (29.2)	76.8 (29.5)	–2.05 (42); .05	0.51; <.001	–10.1 (–78.1 to 57.9)

^a^LoA: limits of agreement.

^b^VF: verbal fluency.

^c^LM: logical memory task.

^d^VPA: verbal paired associates task.

^e^WL: word list.

^f^CP: Constructional Praxis.

^g^TMT-A: trail making test-A.

^h^TMT-B: trail making test-B.

### Language Fluency

#### VF

Participants found all conditions of VF generally comfortable across time points, with animals having a reported comfort index of 82% and 94% across T1 and T2, respectively; supermarket items reported a comfort index of 92% and 92% across both time points, respectively. For words beginning with “F,” a comfort index of 92% was reported for T1, while a comfort index of 78% was reported for T2.

Distributions of VF across each condition in our sample remained constant across time. Normative data were drawn from the Cache County Cohort Study (CCCS) on Memory Health and Aging, including an animal fluency test and the Controlled Oral Word Association Test, the latter being aggregated word recall for 3 letters, F, A, and S [41]. We approximated comparative values for the letter “F” specifically used here by dividing performance for all 3 letter trials by 3 and taking the pooled mean and SD across stratifications reported [52]. Our sample saw higher recall for both phonetic and categorical fluency tasks. Letter fluency was lower than both, which has been seen in other norming studies beyond the CCCS sample used here [56,57]. All conditions of VF maintained significant correlations between 0.50 and 0.73 across time points while also showing no difference in means (Figure 1 [41,52]). Between VF conditions, significant correlations between 0.29 and 0.42 were observed for T1, while significant correlations between 0.26 and 0.52 were seen in T2, in line with norms from past cohort studies [56]. Although bias was generally low, LoA across all conditions were wide, with 95% of the differences between time points expected to fall within 7 to 13 items recalled higher or lower than the average across time points. Notably, the phonetic VF (words beginning with “F”) saw lower total objects recalled than both object VF conditions. Conditions within time points correlated with one another and were all significant.

**Figure 1:**
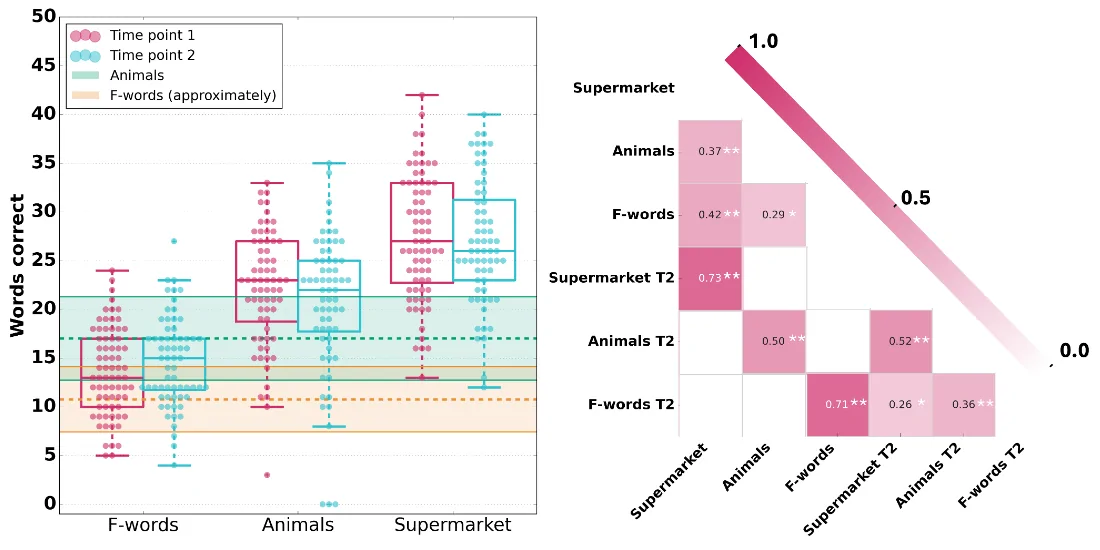
Boxplots and correlations for verbal fluency (VF). The 3 VF categories were words beginning with the letter “F,” animals, and supermarket items. Boxplot midlines show the median; boxes span the 25th to 75th percentiles, and vertical lines span the 10th to 90th percentiles. Comparative means from the age range 65-102 years and comprised 507 participants [52]. Comparative values for words beginning with “F” were approximated from performance on the Controlled Oral Word Association Test [41]. *α=.05; **α=.01.

#### BNT

The BNT had the highest numeric comfort index across both time points (T1=94%; T2=94%). Further, although the BNT was consistent across time points, there were strong ceiling effects. During T1, 40% (25/62) of participants were at ceiling, and during T2, 35% (22/62) of participants were at ceiling (median_T1-T2_=22-22; max_BNT_=23). Compared to normative data from CCCS, which were also near the ceiling, we noticed very little variability in our healthy older adults (Figure 2 [35]). Further, paired *t* tests were nonsignificant, and bias was minimal, which, paired with the significant correlation, leads us to believe there was minimal systematic bias across time points.

**Figure 2:**
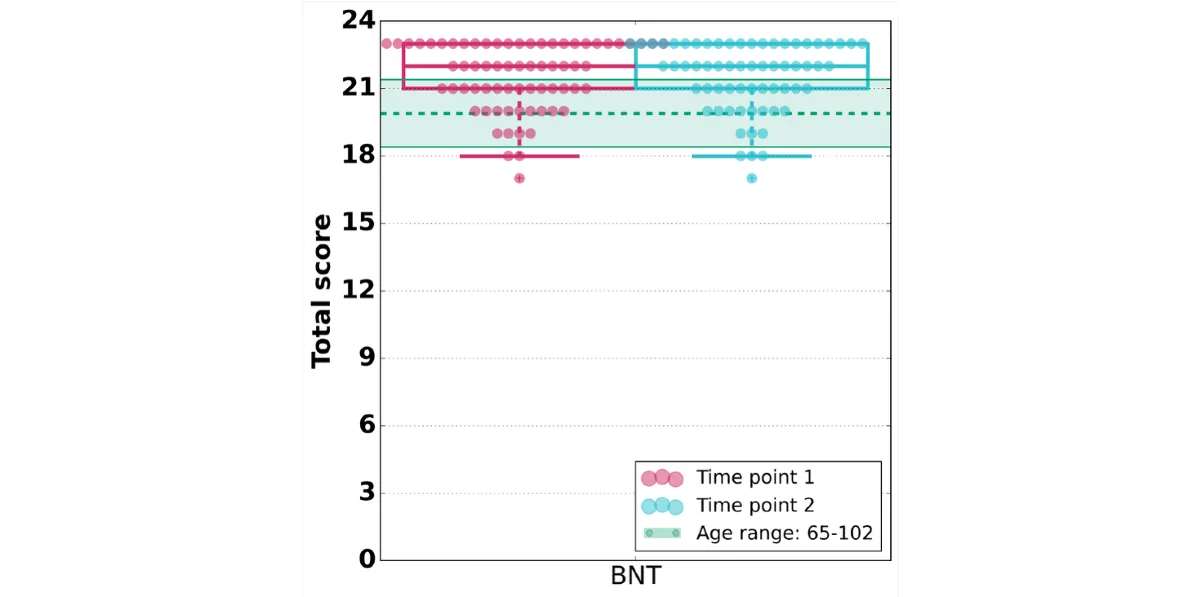
Boston Naming Test (BNT). Boxplot midlines show the median; boxes span the 25th to 75th percentiles, and vertical lines span the 10th to 90th percentiles. Comparative means and SD were calculated by pooling BNT from the 30-item and 15-item versions (age range 65-102 years; n=333) [35].

### Memory

#### Logical Memory

Logical memory was quite tolerable, as during immediate recall, participants’ reported comfort indices for both stories to be high across time points (story A: T1=76% and T2=75%; story B: T1=83% and T2=78%). During T1, delayed recall had a similar comfort index for both stories (story A=86%; story B=89%). Notably, there was a dip in reported comfort indices during T2 story A (70%) compared to story B (80%).

Both conditions of logical memory demonstrated similar distributions of performance across both time points (Figure 3 [52]). Both conditions showed similarly significant correlations as well as nonsignificant *t* tests, showing a stable trajectory and limited between-session variability at the group level. Further, bias was notable, showing that T2 systematically had higher total recall for both conditions. This was confirmed through 1-sample *t* tests comparing the difference in each participant's performance between time points, which, for both stories, was significantly different from 0 for story A *(t*_56_=–2.95; *P*=.005; story B (t_60_=–2.18; *P*=.03). LoA also showed notable within-subject variability for both stories, with total recall varying around 10 items recalled across time points.

**Figure 3:**
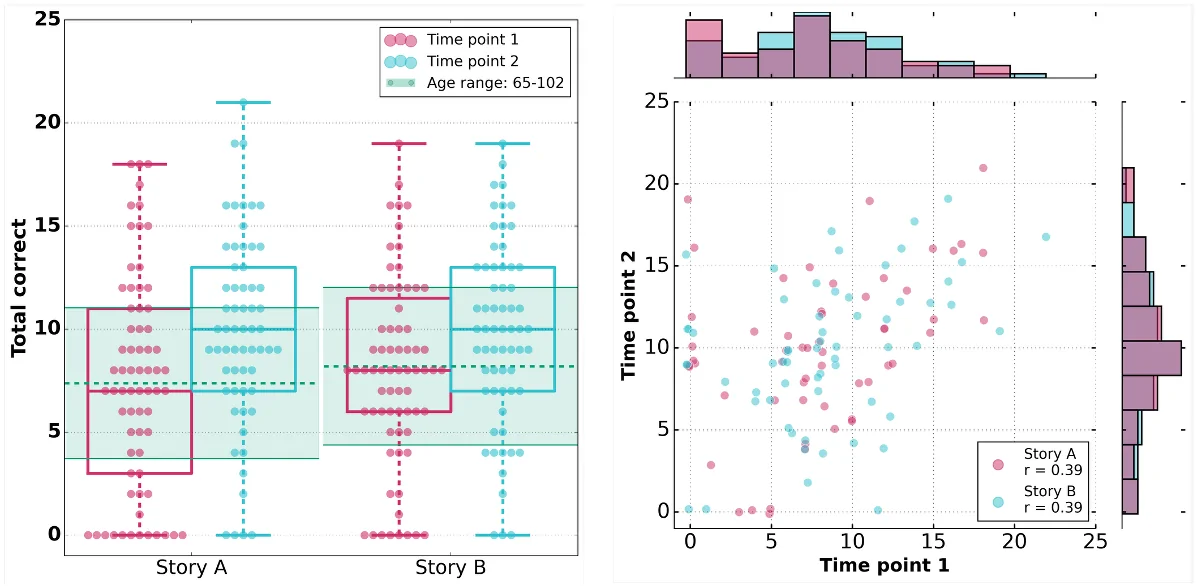
Wechsler Adult Intelligence Scale (WAIS) logical memory story A and story B. Comparative means from the age range 65-102 years and comprised of 507 participants [52]. Left panel shows the boxplots whose midlines show the median and span the 25th to 75th percentiles, and vertical lines span the 10th to 90th percentiles. Right panel shows the distribution across each story, between time points.

#### VPA

The VPA was generally rated with a high comfort index for both during the learning portion (T1=88%; T2=85%) as well as during the delayed recall portion (T1=93%; T2=83%). VPA had notably similar distributions of performance between time points, with a notable improvement in T2, as well as a small ceiling effect (T1-T2: 26%-33% of participants at ceiling; median_T1-T2_=6-7; max_VPA_=8). Moreover, a majority of our sample was comparable to the widely used normative data of older adults (n=156; age 56-89 years) from the oral administration VPA-1 Wechsler Memory Scale-III [44]. VPA delayed recall demonstrated a significant correlation between time points (*r*=0.60; *P*<.001) while also demonstrating a statistically significant *t* test result. This may be due to possible learning effects, as evidenced in the overall improvement of recall in T2 compared with T1. However, both bias and LoA were relatively small, limiting the support for any systematic bias within subjects or between time points.

#### Word List Recall

Word list recall was rated with a high comfort index across time points for the learning portion (T1 = 89%; T2=85%). Delayed recall (T1=89%; T2=86%) also had high comfort indices.

Word list recall also had notably similar distributions between T1 and T2; however, we observed a slight improvement across time with potential ceiling effects across time points (Figure 4 [44,46]; right panel). Aggregated data from 4 different cohorts of cognitively healthy older adults, used as comparative norms here, were comparable to the data collected in this sample, if not slightly lower than our sample [46]. Although the correlation between time points was significant, the *t* test across time points showed significant differences at the group level. Bias was generally low; however, LoA across all conditions were high, with 95% of the differences between time points expected to fall within 2.9 items recalled higher or lower than the average across time points.

**Figure 4:**
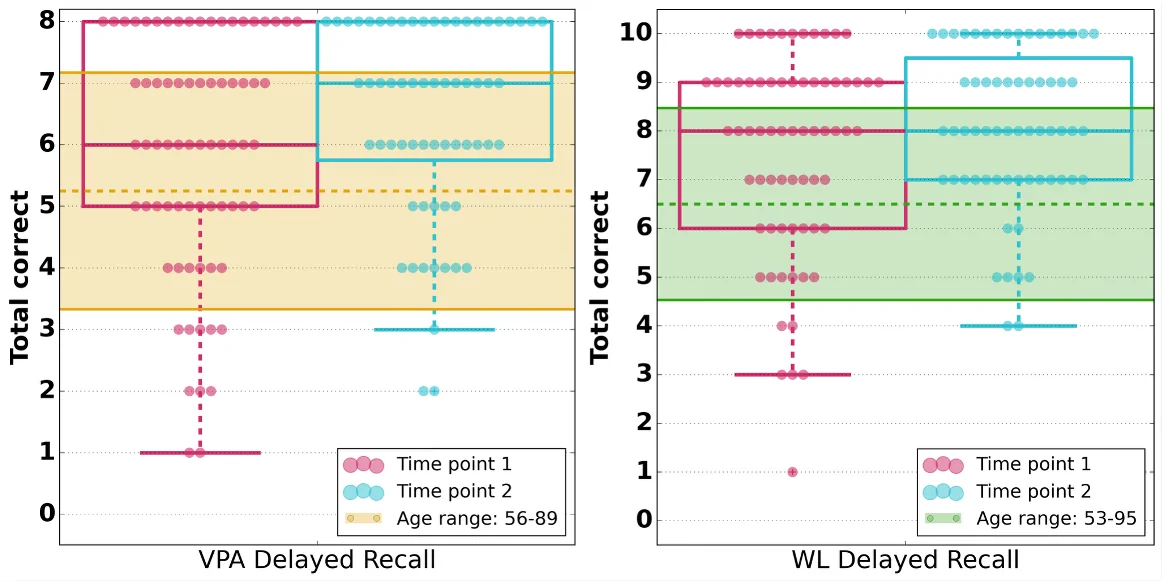
Verbal paired associates (VPA) delayed recall (left panel) and word list (WL) delayed recall (right panel). Boxplot midlines show the median; boxes span the 25th to 75th percentiles, and vertical lines span the 10th to 90th percentiles. Comparative data for VPA are pulled from Wechsler Memory Scale-III (age range 56-89 years; n=156) [44]. Comparative data for WL recall are aggregated across Consortium to Establish a Registry for Alzheimer’s Disease (CERAD) norms, a community cohort of cognitively healthy older adults (age range 53-95 years; n=1662) [46].

### Praxis Memory

#### CP

CP reported high comfort indices across time points, as participants reported an index of 87% and 91% in T1 for immediate copy and delayed recall, respectively. For T2, immediate copy had a comfort index of 89%, while delayed recall was 85%.

CP total recall scores were similar across time points and further showed notable ceiling effects (Figure 5 [47,52,58]; left panel). With a nonsignificant *t* test result along with small bias, there was little effect of systematic bias between time points. However, LoA showed nontrivial within-subject variability, with 95% of participants above or below 7.6 between time points, which was notable considering the maximum score was out of 11. This, combined with the nonsignificant correlation between time points, suggests systematic bias that may be caused by prominent ceiling effects (T1-T2: 37%-40% of participants had maximum scores; median_T1-T2_=9-9; maximum=11).

**Figure 5:**
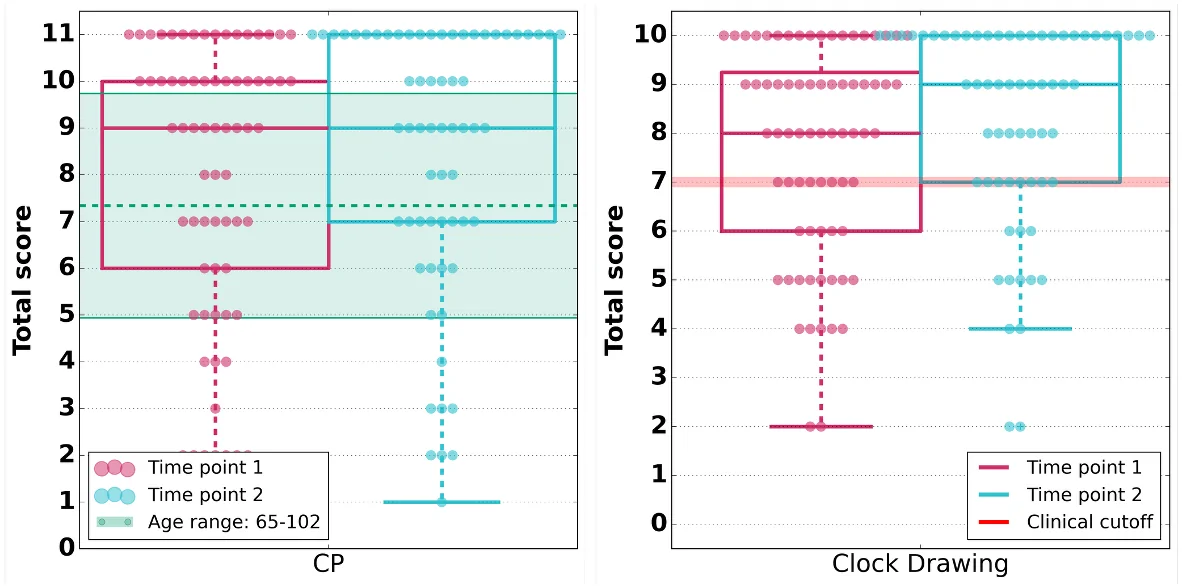
Constructional praxis (CP; left panel) and Clock Drawing Test (right panel). Total score for CP was the sum of all 4 elements participants had to draw. Boxplot midlines show the median; boxes span the 25th to 75th percentiles, and vertical lines span the 10th to 90th percentiles. Normative values for CP have an age range of 65-102 years (n=484) [52]. Clock Drawing Test boxplot shows the commonly used cutoff of 7 [47,58].

#### Clock Drawing

Participants rated the clock drawing task with a comfort index of 90% for T1 and 93% for T2. Clock drawing maintained similar distributions, with ceiling effects across time, and a notable decrease in variability in session 2 compared with session 1 (Figure 5; right panel). Of participants who completed both sessions, 25% (16/63) of participants for T1 had maximum scores, while 40% (25/63) had maximum scores for T2 (median_T1-T2_=8-9; maximum=10). Although there were no significant differences between time points at the group level, correlations were attenuated across time points and were not significant. This may be due to ceiling effects, as bias remained close to 0 and LoA showed acceptable within-subject variability.

### Executive Functioning: TMT

The TMT task was collected on both the PART platform as well as a paper version that was administered remotely; thus, we used the paper draw times as the comparative sample for digital performance. Of note, across either modality across each session, we removed participants who had more than 3 errors or spent more than 160 or 300 seconds for parts A or B, respectively, a commonly used clinical cutoff (n_removedT1_=7; n_removedT2_=13) [59].

For T1, TMT part A and part B reported high comfort indices for both paper (part A=91%; part B=91%) and digital (A=92%; B=87%). For T2, parts A and B, comfort indices were rated similarly, but to a comparatively lesser extent for both paper (part A=82%; part B=80%) and digital (part A=84%; part B=77%).

For TMT part A, distributions of total draw time largely overlapped across modalities and time points, with comparable medians and IQRs observed both within and between modalities. Further, there were no significant differences between means within the paper or digital modalities between time points for either part A or part B. However, within modalities, T1 saw a significant difference for part A (t_52_=–2.16; *P*=.04) but not for part B. T2 saw a significant difference in means for both part A (t_51_=–3.98; *P*<.001) and part B (t_47_=–3.17; *P*=.01). Correlations between time points and across time points were all significant, ranging from 0.35 to 0.66 (Table 1 and the bottom row of Figure 6).

**Figure 6:**
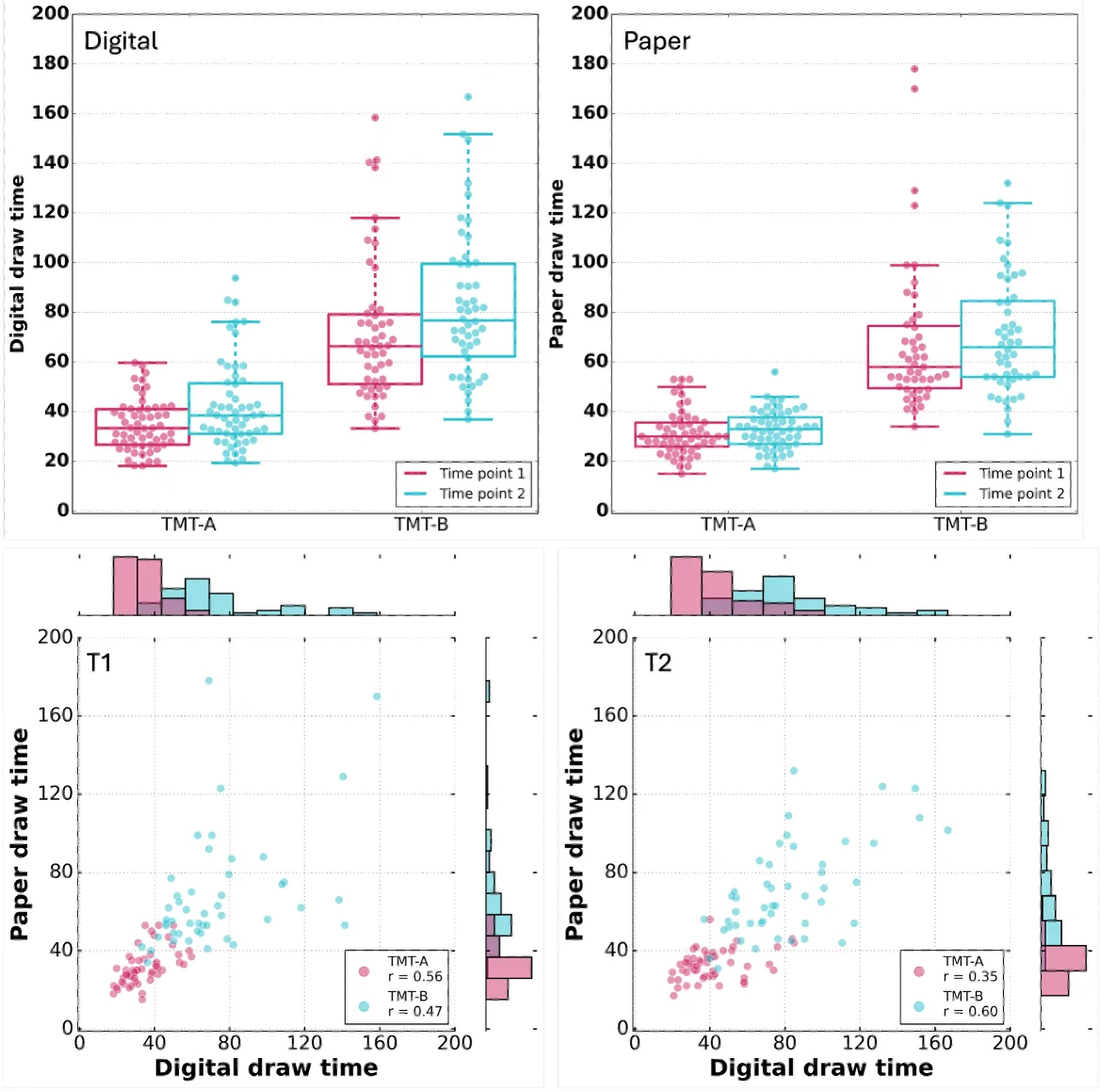
Trail making test (TMT): digital and paper total draw times. Boxplot midlines show total draw times for TMT parts A and B, with different colors denoting different time points. The midlines show the median; boxes span the 25th to 75th percentiles, and vertical lines span the 10th to 90th percentiles (top panels). Scatterplots show the Pearson correlation between TMT modalities across time points (bottom panels).

## Discussion

This study examined the acceptability and short-term reliability of a digital neuropsychological battery administered remotely using the PART app in a cohort of cognitively healthy older adults. Importantly, our sample’s distributions of performance on digital versions of paper-and-pencil tasks were comparable to the ranges found in clinical norming studies, and participant feedback indicated feasibility and self-reported comfort. Below, we explore the implications of these findings and, in turn, discuss the broader contributions of PART to the digital assessment landscape.

The current findings aim to situate PART within the broader landscape of digital cognitive assessment, highlighting unique contributions. PART is a flexible framework for repeated, remote testing across multiple cognitive and perceptual domains within the same ecosystem. This sets a precedent for cross-domain testing which, when further combined with open-access principles, results in a tool capable of richer behavioral phenotyping in digital environments. The configurability of testing schema that combines seemingly disparate constructs has immense promise for multi-domain monitoring of aging. PART is one of many digital tools that are being leveraged by researchers, and future work should aim to harmonize across different software platforms and device types, enabling more direct comparability across studies and settings.

Feasibility was generally acceptable and was consistent with mobile, remote cognitive studies in older adults [60]. Digital TMT may require further investigation, as we excluded a substantial number of individuals using our cutoff criteria (ie, 3 or more errors and total draw times above 160 and 300 seconds for parts A and B, respectively). This could be interpreted as participants struggling with task interfacing, rather than actual cognitive deficits, as all the individuals excluded because of their digital performance would have otherwise not been excluded based on their paper performance, which, in turn, limits the interpretability and generalizability of our TMT reliability estimates. It should be noted that we did see nontrivial participant dropout from screening to consent and consent to T1, possibly due to difficulties in communication and acceptance noted in remote cognitive assessment [61]. Future work should examine strategies to lower overall attrition rate from screening to completion as well as target a larger sample. Stronger retention strategies should be used that account for differential participation based on demographics as well as strategies to increase participants’ perceived value of participation, both shown to affect retention rates [60]. Although the testing was over an hour on average, we did not observe any systematic fatigue effects such as reduced comfortability over the course of testing nor mention of fatigue from participants. Overall, over 75% of participants rated each task as comfortable, with the battery being 88% tolerable during T1 and 84% tolerable for T2 (Table S1 in Multimedia Appendix 1). It should be noted that although retention from T1 to T2 was generally high, our larger study design, which asked for 4 experimental sessions over the course of a week, twice, introduced nontrivial burden.

Several measures demonstrated moderate stability (*r*=0.50-0.73), while others showed lower agreement and substantial within-person variability, notably clock drawing and CP. Bland-Altman LoA showed nontrivial within-subject variability, notably seen in the TMT (part A=–44.7 to 29.2; part B=–78.1 to 57.9). Of note, bias was generally low across tasks but was noted for both logical memory stories (story A=–2.1; story B=–1.5) and TMT (part A=–7.8; part B=–10.1), leading us to believe that the short retest period may have introduced practice effects in these tasks, especially. Further, several participants showed ceiling effects across time points, including clock drawing (11/63, 17% with maximum scores across time points), CP total recall (7/61, 11% with maximum scores across time points), and BNT (16/62, 11% with maximum scores across time points), indicating limited sensitivity for high performers. Although mean scores fell within 1 SD of published norms (eg, CERAD and CCCS), categorical fluency performance (supermarket items and animals) in our sample trended higher, possibly reflecting the high educational attainment of our participants (mean 16.6 years; SD 4.0 years) [62].

The sample was also predominantly female, which may have influenced fluency performance, as some evidence suggests steeper age-related declines in categorical, but not letter, fluency among men relative to women [63], though other studies report minimal sex differences after accounting for education [64,65]. The overrepresentation of women and highly educated individuals limits the applicability of our findings to broader older adult populations, and, further, our sample was not representative of the Southern California populations from which it was drawn. While age distributions were broadly comparable to those of normative samples, these demographic characteristics should be considered when interpreting observed performance levels. Future studies must aim to stratify sampling to capture trends from both individuals with lower educational attainment and those from historically underserved communities who stand to benefit most from accessible cognitive assessment.

These comparisons serve as group-level descriptions of our current sample’s performance on digital tasks compared with generally comparable samples from past work completing paper versions of the same tasks. For example, our comparisons in phonemic VF were approximated based on normative data from 3 letters (F, A, and S), which was a limitation as each individual letter differed nontrivially, and thus, this comparison should be interpreted with caution. The current normative benchmarking approach, while informative at the group level, does not permit conclusions about equivalence at the individual level. Using the TMT evidence in this study as a benchmark, future validation work should involve administering both digital PART versions and traditional paper-and-pencil versions to the same participants in a counterbalanced order, allowing direct within-individual comparisons.

These results imply that while the PART battery can reproduce normative group patterns in a remote setting, care is needed when interpreting change at the individual level over short intervals, not only due to individual differences but especially for tasks susceptible to practice effects or ceiling effects, something observed in the population-based norming studies leveraged in the current work. Of note, our protocol was partially constrained by logistical considerations, such that our time interval may have been too short to prevent practice effects, as evidenced by significant improvements on logical memory and TMT between time points. Promisingly, our sample’s distributions of performance on our task battery across the 2 time points were generally near the distributions in past studies leveraging paper-and-pencil versions of similar tasks. Although there is benefit in rapid and frequent cognitive testing, future validation studies that leverage PART should induce longer retest periods to ensure data avoid practice effects.

Despite positive findings regarding battery acceptance and reliability, our study has several notable limitations. Our sample was predominantly White, educated, and female, which constrains generalizability. Several studies have long identified differences in neuropsychological task performance depending on demographic factors such as race, ethnicity, sex, education, and socioeconomic status [64,66,67]. Importantly, our comparison data were stratified similarly in terms of age and race, yet not biological sex or education. However, future efforts to use these tasks in PART should aim to include a larger population of individuals with lower education and minority backgrounds. A notable limitation of our protocol was its reliance on mailed researcher-configured hardware. While this approach standardizes the testing platform and ensures more consistent data fidelity across participants, it is resource-intensive and not as scalable to large populations compared with using participant-owned devices.

Of note, our work focused on group-level consistency, which was achieved by comparisons with the normative samples along with group-level reliability analyses. Future studies should investigate using inferential statistics if any differences exist at the levels of clinically meaningful changes in digital versions of paper-and-pencil neuropsychological tasks. Further, we collected limited usability data and, rather, investigated self-reported comfort levels. Older adults often have varying levels of digital literacy and comfort with touchscreen devices, factors that could potentially influence test performance [68]. Moreover, physical limitations such as reduced fine motor skills or impaired vision can exacerbate the challenges of engaging with digital interfaces, such as smaller devices and less interactive feature spaces [69]. Although administering a comfort question after each task was informative, this approach is limited insofar as it is not a validated measure and captures only one dimension. Measures of digital literacy and validated user-experience instruments (eg, senior technology acceptance model [70]) were not administered and should be added to future studies, as they remain critical for future successful digital data collection [11,71]. Moreover, many of our tasks required manual scoring, which can introduce subjectivity that can bolster or attenuate reliability and, although all raters were thoroughly trained, future studies should aim to evaluate interrater reliability of scoring in the analytic data pool or investigate the potential for automatic scoring of these tasks using AI [72,73].

Further, alternative strategies of study design and measurement itself could be used, bolstering ecological validity. Although the present study captures participants’ cognitive performance in a more naturalistic setting (ie, at home vs in the laboratory), participants were tested with unfamiliar devices in potentially atypical settings (ie, long Zoom visits) rather than using their own smartphones or tablets in their day-to-day environment. Shorter, more frequent measurement strategies using participants’ own devices could potentially account for context-varying influences such as mood, fatigue, time-of-day effects, or item-level response patterns, better isolating cognitive change from extraneous sources of variability not directly related to task performance [74]. Largely, digital assessment tools that are highly configurable present an opportunity to tailor design around assessments to account for a variety of ecologically constraining factors.

Importantly, many of the tasks examined in the current work may have alternative outcome measures unique to digital modalities, which could afford increased measurement precision and sensitivity, thereby enhancing researchers’ and clinicians’ ability to detect subtle, within-person cognitive changes and fluctuations associated with aging. For example, work validating alternative digital forms of the TMT has identified outcomes beyond draw time, such as time between circles or number of lifts, as highly predictive of other executive functioning measures and global cognitive screeners [49,50]. It should also be noted that although certain stylus-based tasks (eg, drawing lines or tapping responses) show evidence of reliability, issues in interface design and interaction (eg, digital “smudging”) have been identified as key covariates that require more testing, validation, and fine-tuning [49,75]. Future work should further examine this interplay between cognition and digitally derived motor outcomes as well as the degree to which their measurement sensitivity is affected by these extraneous factors.

Future work using PART can and should address the new avenues digital data collection affords. PART is available on public platforms for desktop, smartphone, and tablet devices (eg, iOS App Store and Google Play Store), allowing for repeated, frequent testing, which may be more sensitive in capturing cognitive changes [28]. Our battery was administered with oversight from trained research administrators, which is both a feature insofar as it may improve data quality and most closely mirrors clinical practice, and a limitation, as fully self-administered batteries may be more scalable. Future work should examine the degree to which these tasks could be fully self-administered to older adult participants on their own devices. Another key advantage of digital assessment platforms is the potential to replace or supplement ceiling-prone tasks with adaptive or more sensitive paradigms [76]. Future studies using PART can incorporate adaptive item difficulty (eg, expanding BNT item sets, using more complex drawing tasks, or adding more challenging executive function conditions) to improve sensitivity and reduce ceiling effects in healthy older adult cohorts.

PART also collects participant audio recordings and temporal tablet touch data, which may provide alternative outcome measures as valuable, or more valuable, when compared to traditional neuropsychological test outcomes. All our nondrawing tasks in the current work involved recording speech and may provide critical information that improves upon measures originally designed under the constraints of paper-and-pencil tasks [11]. Although these speech analyses were out of the scope of the current work, future analyses should investigate how these speech recordings can act as a primary or supplementary data stream, as recent work has shown its utility in categorizing patients with mild cognitive impairment and Alzheimer disease [77,78].

In summary, this study demonstrates that a remotely administered digital neuropsychological battery using PART is feasible, well-tolerated, and produces acceptable short-term reliability in older adults within expected ranges compared to well-established paper-and-pencil counterparts. It advances longitudinal aging measurement through a flexible approach and supports remote screening, longitudinal monitoring, and inclusion of otherwise excluded participants. PART complements in-person assessment while providing potential avenues to expand the capabilities of traditional assessment. Critically, its open-access, configurable framework positions it as a tool that can serve both clinical screening and basic research aims, adaptable across domains and populations. Future research should take advantage of remote assessment frameworks through larger, more diverse sampling procedures, explore the potential for more ecologically valid data schemas, and consider evaluating reliability and feasibility in clinical cohorts.

## Appendix

Multimedia Appendix 1 Flowchart detailing data cleaning and participant retention alongside self-reported task comfort across testing sessions.

## Abbreviations

ARC: Ambulatory Research in Cognition
CANTAB: Cambridge Neuropsychological Test Automated Battery
CCCS: Cache County Cohort Study
CERAD: Consortium to Establish a Registry for Alzheimer’s Disease
LoA: limits of agreement
M2C2: Mobile Monitoring of Cognitive Change
MCAR: missing completely at random
NIH: National Institutes of Health
PART: portable automated rapid testing
TabCAT: Tablet-Based Cognitive Assessment Tool
T-MoCA: Montreal Cognitive Assessment–Blind/Telephone
TMT: trail making test
UC: University of California
VF: verbal fluency
VPA: verbal paired associates

## Data Availability

The datasets generated or analyzed during this study are available from the corresponding author on reasonable request.
