# Supplementary material for: Neuropsychological Assessment Using Portable Automated Rapid Testing in Cognitively Healthy Older Adults: Test-Retest Reliability Study
Source: JMIR Form Res. 2026 Sep 23;10:e90646. doi: 10.2196/90646 (PMC13646780; doi:10.2196/90646)
Supplement: Supplementary file 1 [file formative_v10i1e90646_app1.docx]

**Figure S1.** Flowchart of data cleaning procedure. Boxes show N participants at each stage: screened, had ≥1 raw measurement from any task, removed participants with <50% usable data per session after outlier removal and data cleaning, removed participants with <70% usable data across both sessions, and included in the final analysis. Outlier removal = values >3 SD from session mean. 2 participants were removed from the T1 data pool due to a software glitch in PART that resulted in NaN’s in the raw data. 6 participants were removed from T2 due to an administration error where the administrator incorrectly configured PART, resulting in errors in recorded participant responses. 1 participant was removed due to more than 70% missingness across both sessions, which included a PART software error that resulted in negative draw time values for the TMT. Numbers are computed from the dataset and reflect all cleaning steps applied in the analysis pipeline.

**Table S1.** Self-reported comfort of participants. Participants were asked two yes or no

questions, “Were you comfortable during the last task?”. Percentages represent Total “Yes”

responses + 0.5*Total “Maybe” responses / Total. VF = Verbal Fluency; LM = Logical

Memory; BNT = Boston Naming Task; VPA = Verbal Paired Associates; WL = Word List;

CP = Constructional Praxis; TMT = Trail Making Task.

|  | **T1 (N = 53)** | | **T2 (N = 51)** | |
| --- | --- | --- | --- | --- |
|  | **Yes/No/Maybe** | **Comfort (%)** | **Yes/No/Maybe** | **Comfort (%)** |
| VF: Animals | 43/3/1 | 82.1 | 47/0/2 | 94.1 |
| VF: Supermarket | 48/1/1 | 91.5 | 46/0/2 | 92.2 |
| VF: Letter F | 48/0/2 | 92.5 | 38/6/4 | 78.4 |
| LM: Story A: Immediate recall | 38/5/5 | 76.4 | 36/7/4 | 74.5 |
| LM: Story A: Delayed recall | 45/3/1 | 85.8 | 34/2/3 | 69.6 |
| LM: Story B: Immediate recall | 43/5/2 | 83.0 | 38/6/4 | 78.4 |
| LM: Story B: Delayed recall | 47/3/0 | 88.7 | 40/1/2 | 80.4 |
| BNT | 50/0/0 | 94.3 | 48/0/0 | 94.1 |
| VPA: Learning 1 | 39/3/6 | 79.2 | 40/7/1 | 79.4 |
| VPA: Learning 2 | 49/0/0 | 92.5 | 42/3/2 | 84.3 |
| VPA: Learning 3 | 48/0/2 | 92.5 | 46/1/1 | 91.2 |
| VPA: Recall | 49/1/0 | 92.5 | 42/0/1 | 83.3 |
| WL: Learning 1 | 42/3/4 | 83.0 | 38/6/3 | 77.5 |
| WL: Learning 2 | 47/0/2 | 90.6 | 44/2/1 | 87.3 |
| WL: Learning 3 | 49/0/0 | 92.5 | 46/1/0 | 90.2 |
| WL: Delayed Recall | 47/1/0 | 88.6 | 44/2/0 | 86.2 |
| CP: Immediate recall | 46/1/0 | 86.8 | 45/1/1 | 89.2 |
| CP: Delayed recall | 47/0/2 | 90.6 | 43/0/1 | 85.3 |
| Clock Drawing | 47/2/1 | 89.6 | 47/0/1 | 93.1 |
| TMT-A: Paper | 48/0/0 | 90.6 | 42/0/0 | 82.4 |
| TMT-A: Digital | 48/0/1 | 91.5 | 43/0/0 | 84.3 |
| TMT-B: Paper | 48/0/0 | 90.6 | 41/0/0 | 80.4 |
| TMT-B: Digital | 45/2/2 | 86.8 | 37/2/4 | 76.5 |
